# ID 323 - Treze Anos de Conitec: incorporação de tecnologias e monitoramento

**DOI:** 10.5327/2237-9622.2025.v34s1.323

**Published:** 2025-11-25

**Authors:** Erika Akemi Tsujiguchi Bernardi, Maykon Andersom Pires de Novais

**Keywords:** avaliação da tecnologia biomédica, acesso a medicamentos essenciais e tecnologias em saúde, Sistema Único de Saúde

## Abstract

**Introdução:**

Desde sua criação em 2011, por meio da Lei n.º 12.401, de 28 de abril de 2011, a Comissão Nacional de Incorporação de Tecnologias no Sistema Único de Saúde (Conitec) vem desempenhando um importante papel na avaliação para a disponibilização de tecnologias em saúde, assessorando o Ministério da Saúde. Ao passo que as tecnologias em saúde são disponibilizadas, é importante o monitoramento delas, realizado pelo Monitoramento do Horizonte Tecnológico (MHT), para dessa forma entender o quanto elas estão sendo benéficas para a população. Este trabalho tem como objetivo avaliar o volume de tecnologias discutidas e incorporadas e quantas destas estão sendo monitoradas pelo MHT após a incorporação.

**Método:**

Foi realizado levantamento das tecnologias demandadas e dos dados do MHT no site da Conitec entre 2012 a 2024. São publicados dados de tecnologias já avaliadas ou em avaliação. Para o total de tecnologias, não foi incluído nenhum filtro. Para obtenção dos dados sobre medicamentos, foi aplicado um filtro para medicamento em "tipo de tecnologia". Esses dados foram analisados em planilha eletrônica. A informação sobre os demandantes foi organizada em quatro categorias (associações, governo, indústria e justiça). O status foi classificado em análise, consulta pública, encerrado, excluído, incorporado, não excluído, não incorporado e restrição de uso.

**Resultados:**

Foram identificadas 1.064 demandas avaliadas pela Conitec que estão divididas em medicamentos (822), procedimentos (168) e produto (74). Os principais motivo para submissão foram incorporação (687) e exclusão (83). Os cinco temas mais presentes nas submissões foram: infectologia (121), reumatologia (84), oncologia (82), hematologia (66), neurologia (61). Os principais demandantes foram o governo (463) e a indústria (310). Com relação ao desfecho das avaliações das demandas, foram incorporadas 332 tecnologias, 257 não foram incorporadas, 84 tiveram o processo encerrado, 78 tecnologias foram excluídas, 58 em análise, 9 estava em consulta pública, 3 após avaliação não foram excluídas, e 1 tecnologia teve restrição de uso. Quanto ao monitoramento pelo MHT, foram 4 medicamentos e 2 avaliações de desempenho de 4 medicamentos.

**Conclusão:**

Em 13 anos de Conitec, foram muitas avaliações possibilitando que 332 tecnologias estivessem disponíveis para a população atendida pelo Sistema Único de Saúde (SUS). O principal demandante foi o governo, sendo a Secretaria de Ciência, Tecnologia e Inovação e do Complexo Econômico-Industrial da Saúde (Sectics/MS) responsável, somando 227 demandas. No entanto, quando se analisa o total de demandas de medicamentos incorporados (332) versus a quantidade dessas que foram monitoradas pelo MHT (oito medicamentos), há um volume baixo de monitoramento. Analisando um dos relatórios do MHT (adalimumabe para hidradenite supurativa), conclui-se o quanto é importante esse monitoramento, visto que é nesse momento que se comparam os dados pré e pós-incorporação, possibilitando identificar tendência de aumento ou redução da população prevista, se os resultados estão em linha com os Protocolos Clínicos e Diretrizes Terapêuticas (PCDT). Ainda é possível identificar se o preço praticado pós-incorporação está de acordo com o que foi proposto. Dessa forma, por meio da análise pós-incorporação pelo MHT, é possível identificar tendências e corroborar a tomada de decisão ou a elaboração de estratégias para que as tecnologias disponíveis sejam efetivas, seguras e relevantes para a população.

